# Results of arthroscopic treatment of chondral delamination in femoroacetabular impingement with bone marrow stimulation and BST-CarGel^®^

**DOI:** 10.1051/sicotj/2017031

**Published:** 2017-08-07

**Authors:** Mahmoud Tahoun, Tamer Aly Shehata, Inmaculada Ormazabal, Jesús Mas, Javier Sanz, Marc Tey Pons

**Affiliations:** 1 iMove traumatologia, Hospital Mi Tres Torres Barcelona Spain; 2 Hip Unit, Department of Orthopaedics, Hospital del Mar, UAB Barcelona Spain; 3 Department of Orthopaedics, Menoufia University Al Minufya Egypt; 4 Department of Orthopaedics, IHR Kingdom of Bahrain; 5 Department of Radiology, Hospital Universitari Dexeus Barcelona Spain; 6 Department of Orthopaedics, Clínica Vistahermosa Alicante Spain

**Keywords:** Hip arthroscopy, Cartilage, Bone marrow stimulation, Femoroacetabular impingement, Chondral delamination

## Abstract

*Objectives*: The purpose of this study is to show the preliminary results of using chitosan-based scaffold (BST-CarGel^®^) with microfracture for treatment of acetabular chondral delamination associated with femoroacetabular impingement.

*Methods*: A prospective study was performed on 13 hips. Patients were selected in the age group between 18 and 50 years. Patients with delamination of acetabular cartilage associated with femoroacetabular impingement received arthroscopic debridement and microfracture technique. Then cases with defect > 2 cm^2^ were considered for the application of BST-CarGel^®^ and included in the study. Also, reattachment of the torn labrum and resection of the cam deformity were performed according to the case. For evaluation of the functional outcome, the patients had completed the hip outcome score (HOS) pre- and post-operatively. For evaluation of the regeneration of the cartilage, delayed gadolinium-enhanced MRI of cartilage (dGEMRIC) was used and the percentage of defect filling and type of cartilage studied.

*Results*: Patients had a mean age of 41 years, with moderate to high level of activity (mean Tegner scale 7). The mean size of the chondral defect after debridement was 3.7 cm^2^. The mean HOS for daily live activities has been improved from 64.4 to 87.4 and for sports subscale from 35.2 to 75.2, which is statistically highly significant. All patients had > 90% of filling of chondral defect.

*Conclusion*: The use of BST-CarGel^®^ with microfracture for treatment of acetabular chondral delamination associated with femoroacetabular impingement can improve the functional outcome at two years, with a complete restoration of the cartilage defect in magnetic resonance images (MRI) with specific cartilage sequences.

## Introduction

Femoroacetabular impingement (FAI), as a change in the morphology of the proximal femur or the acetabulum, produces a mechanical disturbance in the hip joint which can initiate the degenerative process and finally osteoarthritis (OA) [1]. There are two serious problems associated with FAI, chondral delamination which occurs more frequently with the non-spherical head of femur (cam type) because of repeated shear forces, and labral detachment, associated to shear forces in cam-type FAI, or damage which is more frequent with the acetabular overcoverage (pincer type) due to repeated compressive forces [1, 2].

Cartilage damage with FAI occurs mainly in the anterosuperior area of the acetabulum and can take the form of fibrillation, delamination, or complete defect, so it is a challenging problem as regards evaluation and management. The imaging techniques have been advanced to help surgeons for a more accurate assessment. The quantitative magnetic resonance imaging (MRI) of the cartilage, as delayed gadolinium-enhanced MRI of cartilage (dGEMRIC) and T2 mapping, can map the concentration of glycosaminoglycans (GAGs) in the cartilage, so it provides high sensitivity and accuracy for detecting early damage and for follow-up of patients after conservative hip surgeries [3, 4].

The articular cartilage has a poor intrinsic healing capacity, so several techniques have been developed to potentiate the cartilage healing and reproduce a new tissue with structural and biomechanical properties similar to those of the normal cartilage [5]. Debridement and microfracture, as easily performed and cost-effective techniques, are considered standard methods for small full-thickness defect [6].

Research efforts have been directed to augment the biochemical properties of fibrocartilage and increase its content of collagen type II and proteoglycan to be more hyaline-like tissue. Chitosan-glycerol phosphate blood scaffold is used to augment the clot formed in the microfractured area and provide more stable environment for growth and differentiation of hyaline cartilage. BST-CarGel^®^ (Piramal Life Sciences, Quebec, Canada) is a novel Chitosan-based implant that is used in experimental studies on animal models, increased stabilization of repair tissue by rapid solidification, inhibiting the retraction of fibrin network and proper integration with the subchondral bone [7, 8].

These researches provided a scientific base for clinical trials in the knee joint to justify the surgical technique of application [9], and reviewing all these previous works encouraged us to carry out this study to show the results of using BST-CarGel^®^ with microfracture in arthroscopic management of patients with acetabular cartilage damage [10, 11].

## Material and methods

### Patient selection

The study population was limited to patients who were diagnosed to have chondral lesion associated with cam- or mixed-type FAI. The preoperative evaluation of all patients was done for clinical diagnosis of FAI by typical pain presentation (C-sign) and physical examination according to an accepted protocol [12]. The radiological assessment was done by X-ray of the pelvis and both hips in anteroposterior pelvic and hip Dunn views, and then measurements were obtained to identify the cam and pincer lesions, as well as despite hip dysplasia (alpha angle, lateral center edge angle, head-neck offset, and acetabular index). Articular cartilage and labral injuries were assessed by magnetic resonance (MR) arthrography. The study was further limited to patients between 18 and 50 years old with no radiological signs of osteoarthritis (Tönnis 0 or 1) or dysplasia (Wiberg < 25°, Tönnis angle > 10°).

During the arthroscopic procedure, size of the chondral lesion was measured after debridement by arthroscopic calibrated probe or ruler. Cases with circumscribed cartilage defect measuring > 2 cm^2^ were considered for the application of BST-Cargel^®^ after microfracture. Thirteen cases were included in this study by fulfilling these criteria. All subjects who participated in the initial attempt were asked to provide a written informed consent prior to study activities to be part of this extension study and after fulfilling the rules of Ethical Committee of Clinical Research.

### Surgical procedure

The full surgical technique was previously reported [10], and by these rules, hip arthroscopic surgery was performed with the patient placed in the supine position on a traction table, and standard anterolateral and distal mid-anterior portals were used as the viewing portal and working portal, respectively. Delaminated cartilage was adequately removed by curette and motorized shaver, until reaching a stable and well-defined margin of healthy cartilage, then removing the calcified layer to expose the subchondral bone. Microfracture was performed by 60° arthroscopic awl with a depth of 2–3 mm and an interval of 5 mm distance until covering the entire defect. Then according to their condition, pincer and labral lesions were treated by acetabuloplasty and refixation of the labrum with three or four suture anchors (Osteoraptor, 2.3 mm; Smith & Nephew). After that, traction was released to access the peripheral compartment, and extended T-capsulotomy may be required for easier manipulation, and cam deformity was removed by motorized burr and with the assistance of fluoroscopic image intensifier.

**Figure 1.**
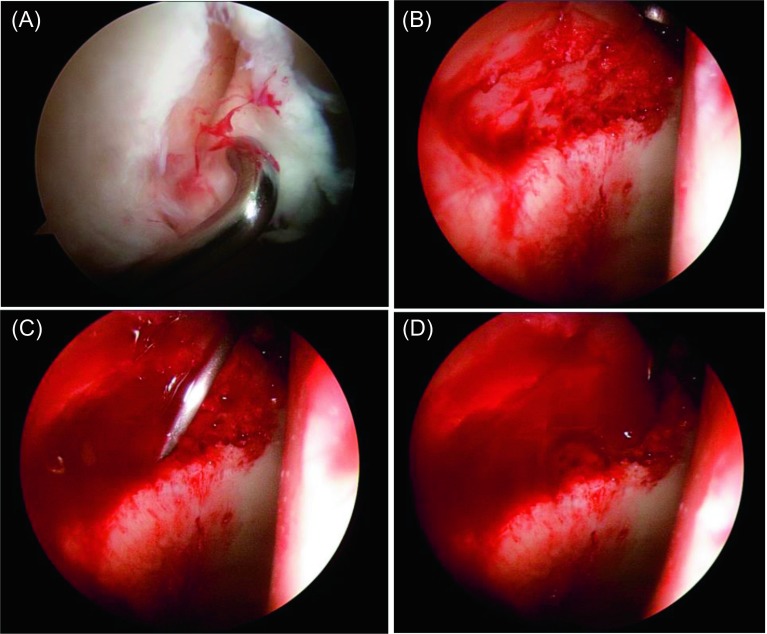
(A) Arthroscopic image of right hip joint showing acetabular chondral delamination before debridment. (B) Chondral defect after debridment and microfracture with bleeding from the penetration holes. (C) Application of BST-CarGel^®^ mixture by 18-gauge needle. (D) Covering of the whole defect with BST-CarGel^®^ mixture.

Capsulotomy was partially closed and BST-Cargel^®^ was prepared according to the manufacturer’s instructions. The traction was reapplied and all the fluid inside the joint was drained using swabs on the lesion surface. The mixture was then delivered in a dropwise manner using a large bent 18-gauge needle making layer by layer until the defect was covered (see Figure 1). The implant was left in place for 15 min for consolidation then the traction was released.

### Postoperative rehabilitation

The patient is encouraged for passive motion from the first day aided by simple analgesia and recommended for six weeks of partial weight bearing (<20 kg) assisted by crutches. With the observation of a physiotherapist, low-contact physical activities can be allowed in the third month, whereas high impact sports must be avoided during the first year after surgery.

### Study group

We prospectively followed 13 patients (see Table 1) who underwent hip arthroscopy for therapeutic treatment of hip pain. None of them have been lost to follow-up. The follow-up for this study was 24 months. There were 10 male patients and three female, with a mean age at index operation of 41 years (25–50 years). Any intraoperative cartilage lesion was measured with an arthroscopic calibrated probe and the mean was 3.7 cm^2^ (3–6 cm^2^). All patients were assessed clinically and completed the hip outcome score (HOS) with subscales for daily activities and sports-related activities pre- and post-operatively [13]. Also, radiological evaluation was done by postoperative X-ray to measure the correction of the alpha angle. Patients were investigated by dGEMRIC after 18–24 months from the surgery to assess the integrity and degree of incorporation of the repair tissue with the surrounding cartilage.

**Table 1.** Baseline parameters of the patients participated in the study.

Number of cases (*n*)	13
Male	10
Female	3
Age (mean ± *SD*)	41 ± 7.7
Onset of symptoms (mean ± *SD*) per months	36 ± 8.2
Tegner level (mean ± *SD*)	7 ± 1.3
FAI	
Cam (*n*)	8
Mixed (*n*)	5
Size of the lesion (mean ± *SD*) in mm	3.7 ± 0.8

We used paired Student’s *t*-test for statistical analysis. Values for *p* < 0.05 were regarded as significant.

## Results

**Figure 2.**
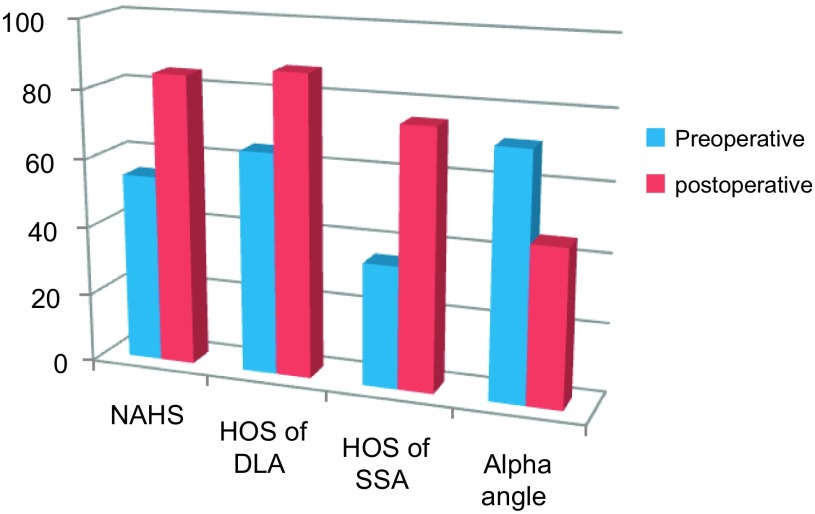
Chart illustrating the change in functional scores and alpha angle from preoperative to postoperative.

The pre-operative diagnosis was cam-type FAI in five cases and mixed (cam and pincer) type in eight. In all cases, labral reattachment was needed. The HOS for daily live activities pre-operatively was 64.5 (95% confidence interval (CI) 42.1–89.5) and for sports 35.3 (95% CI 12.5–55.5). At 24 months postoperatively, the mean HOS for daily activities had improved to 87.4 (95% CI 57.9–100), a difference which was statistically highly significant (*p* < 0.01). The mean HOS for sports had improved to 75.2 (95% CI 41.6–100), which was also statistically highly significant (*p* < 0.01). Figure 2 shows these results.

**Figure 3.**
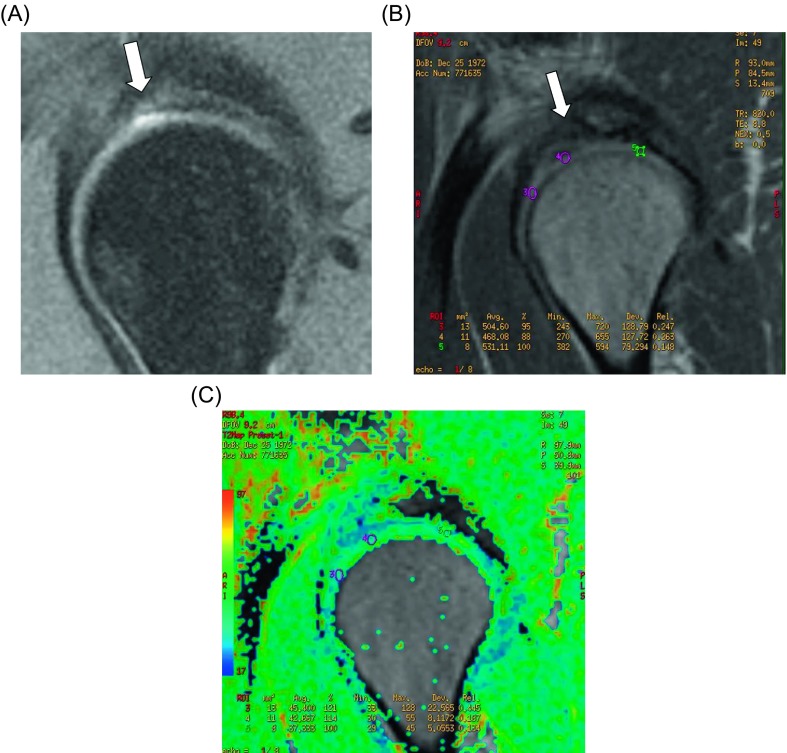
Pre- and post-operative MR of case number 1. The improvement is evaluated quantitatively (100% of filling of the lesion) and quantitatively (decrease in T2 values). (A) PDFS sagital imaging of the right hip showing the absence of cartilage in area of the anterosuperior articular surface of the acetabulum. (B, C) T2 mapping of the same hip 18 months after the surgery and the repair of the ulcerated area with improvement (decrease T2 values, showing mixed fibrocartilage and hyaline cartilage).

**Figure 4.**
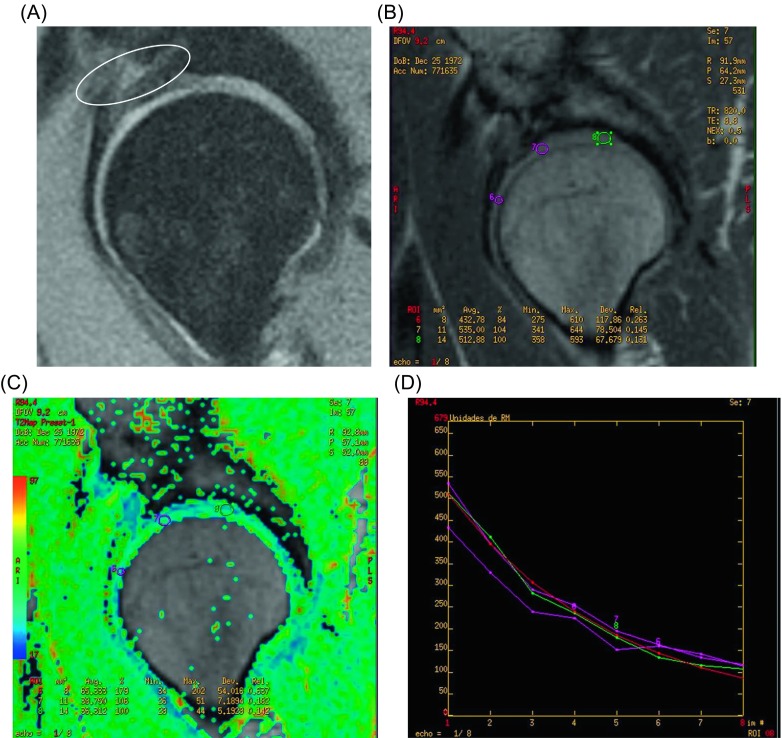
Pre- and post-operative MR of case number 2. The improvement is evaluated quantitative (100% of filling of the lesion) and qualitative (decrease in T2 values, showing hyaline cartilage). (A) PDFS sagital imaging of the right hip showing the extensive zone of alteration of the signal and delamination in the anterosuperior quadrant of the acetabular articular surface. (B–D) T2 mapping of the same joint 15 months after the surgery and the morphological improvement of the chondral lining and practical normalization of T2 values.

The femoral head sphericity was achieved in all patients. The alpha angle improved from 71° (95% CI 63–80) to 45° (95% CI 40–52). The dGEMRIC at 18–24 months postoperatively was done in 10 cases and showed 100% of filling of the defect in eight (80%) and 90% in two (20%). The repaired tissue was fibrocartilage in five cases (50%), hyaline cartilage in three (30%), and mixed in two (20%). In Figures 3 and 4, examples of mixed cartilage and hyaline cartilage regeneration are shown.

## Discussion

The microfracture technique has been extensively studied for a long time as a cost-effective and less invasive method for treatment of cartilage lesions, and became a gold standard of care especially for small full-thickness defects supported by the excellent early functional improvement. The microfracture technique depends on the stimulation of subchondral bone marrow by penetrations, which liberates undifferentiated stem cells, and a blood clot is formed in the defect which provides a supporting environment for the cartilage progenitor cells, and finally differentiates into stable fibrocartilage. But in the case of a large defect, the fibrocartilage patch becomes more unstable and more liable to detach because of clot retraction [7]. Long-term studies of patients treated with microfracture for knee cartilage defects showed a clear decline in the functional outcome after two years, although average knee function scores remain above preoperative levels. The reason for this functional deterioration can be related to poor filling volume of repair cartilage or the inferior wear characteristics of the fibrocartilaginous repair tissue resulting from marrow stimulation [14, 15]. In our series, functional outcomes showed an improvement of clinical outcomes but unlike the knee study, with a high filling volume in repair cartilage.

Since the process of tissue repair depends on the organization of the initial blood clot, potentiating this clot by biological scaffold can increase the integrity of the repair tissue. Chitosan is a positively charged polysaccharide polymer that can adhere to the negatively charged tissue surfaces including cartilage, so it can be considered a biocompatible scaffold with negligible immunogenicity. Based on these experiments, animal studies were done to demonstrate the effect of the application of chitosan-based scaffold on the microfractured cartilage defect. It was concluded that the resultant repair tissue showed significant filling volume and proper integration into the surroundings, and there was an increase in the content of glycosaminoglycans, more organized collagen type II and higher cell density [7, 8]^.^

A randomized controlled trial used BST-CarGel^®^ as a scaffold material with microfracture to treat chondral lesions in the knee joint, and showed significant improvement in the quantity and quality of the repair cartilage at 12 months in patients with full-thickness cartilage lesions compared with microfracture treatment alone. Also, this study demonstrated that percent of filling (%fill) following BST-CarGel^®^ treatment was not affected by the size of the lesion compared to lesions treated with microfracture alone which showed a decrease in %fill with increase in defect size. T2 relaxation time was used as a parameter to assess the quality of the repair cartilage and showed significant difference between BST-CarGel^®^ treated cartilage and microfracture alone indicating improved quality of repair cartilage, although not yet at the level of normal cartilage [16]. Clinical improvement occurred in both groups of patients which was equivalent at 12 months and was significantly above the baseline. Furthermore, extension of this study for five years follow-up showed similar comparable results between the two groups as those of the first year regarding the repair cartilage quantity and quality by %fill and T2 relaxation time [9]. The more interesting finding in this extended trial is the equivalent functional outcome observed in both groups after five years measured by The Western Ontario and McMaster Universities Osteoarthritis Index (WOMAC) score although some parameters, such as higher baseline body mass indices (BMIs) and larger lesion volume, were against the BST-CarGel^®^ treated patients while lower ages and shorter time from the onset of symptoms could be advantages for BST-CarGel^®^. Also, the study attributed the good functional outcome in patients treated with microfracture alone to the strictly obeyed rules and optimally performed steps of the technique.

Current standard diagnostic tools for the assessment of cartilage are characterized by a number of limitations – they are merely qualitative and user-dependent, thus limiting intra-method sensitivity, specificity, and reliability as well as intermethod comparability.

Magnetic resonance imaging (MRI) is the current reference imaging modality for the non-invasive joint and cartilage assessment. Although substantial efforts have been made over the past years to establish MRI biomarkers for cartilage degeneration, these methods have not made their way into clinical practice [17].

Therefore, the demand for reproducible and consistent decision-making had led to the development of quantitative MRI techniques.

Although the exact biophysical and pathophysiological relations of these approaches require further investigation, their overall correlation with reference histology is well proven, in particular for dGEMRIC, T1rho, and T2.

Quantitative T2 maps were extracted from multi-echo MR data with a mono-exponential fitting routine. T2 mapping has been taken up rapidly in recent years and is now commercially available for many 1.5T and 3T scanners. There is a strong relationship between increasing T2 mapping signal and the histological severity of early OA. The signal is also greater in the cartilage of patients with radiographic OA than in healthy controls. T2 mapping may be less sensitive than dGEMRIC in detecting early degenerative change. However, it is a more universally accessible, more standardized, and more comparable method, being the one of more easy and affordable realization in almost all the equipment of 1.5 or 3T [18].

T2 relaxation times in FAI patients have shown increased heterogeneity in the anterior acetabular region and increased relaxation time as compared to controls.

The evolutionary follow-up of the treated chondral lesions requires the realization of T2 mapping scans with the same technical conditions, in the same device of MRI, and with the same protocol of acquisition and segmentation of the images, since the values obtained of T2 mapping signal are not absolute, but relative, depending on the mentioned parameters.

In our prospective study, 13 cases with FAI associated with acetabular chondral delamination were treated with microfracture and BST-Cargel^®^ and were observed with a mean follow-up of 31 months; during this period, all of them showed significant improvement in the functional outcome scores HOS of daily life activity and sports activity (Table 2). The mean age of these patients was 41 years at the time of surgery, and they were adapted to the activity level, moderate to high, on Tegner scale with mean of seven before complaining of hip problems, and it is considered a point of strength in this study because it is difficult to achieve detectable improvement in the function of such patients. After adequate debridement, the mean size of the chondral defect was 3.7 ± 0.8 cm^2^, and there is a significant association between these chondral lesions and labral tear as all cases needed labral reattachment, and this was expected to be found with the presence of cam deformity diagnosed in all the cases preoperatively.

**Table 2.** Comparison between preoperative and postoperative values (mean ± *SD*) of functional scores and alpha angle.

	Preoperative	Postoperative	*p* value
HOS of DLA	64.5 ± 16	87.4 ± 18	*p* < 0.01
HOS of SSA	35.3 ± 19	75.2 ± 29	*p* < 0.01
Alpha angle	71.3 ± 5.9	45.6 ± 3.4	*p* < 0.01

As a part of the management procedure, femoroplasty was done with adequate resection of the cam deformity and evaluated by comparing mean preoperative (71.3 ± 5.9) and postoperative (45.6 ± 3.4) alpha angles showing significant correction (Table 2).

The limitations of this study are the small number of population and three of the patients could not make it to the dGEMRIC study because of financial and logistic obstacles and lack of control group. Also, these results alone cannot conclude that BST-Cargel^®^ application is the principal factor leading to such an improvement in the functional scores because the high level of activity (mean 7 Tegner score) and relatively short preoperative symptomatic period (mean 36 months ± 8.2) and well-performed microfracture technique are important contributing factors, so continuation of follow-up for longer period and analysis of the repair cartilage tissue by quantitative and qualitative methods of radiological investigation are required to give a final conclusion.

In summary, the use of BST-Cargel® as a scaffold material after microfracture can maintain a good functional outcome in patients with FAI associated with acetabular chondral delamination for more than two years with necessary radiological evaluation for the quantity and quality of the repair cartilage tissue.

## Conflict of interest

The authors report that they have no financial conflict of interest in connection with this article.
